# Summer Showers

**Published:** 1871-07

**Authors:** 


### SUMMER SHOWERS

Frequently overtake persons and "wet them to the skin;" it is then safer to walk steadily and rapidly on, until the clothes become dry again, than to stop under a shelter and remain there still until the storm is over. If home is reached while the clothing is yet wet, take some hot drink instantly, a pint or more; go to the kitchen fire, remove every garment, rub the whole body with a coarse towel or flannel, put on woolen underclothing, get into bed, wrap up warm, and take another hot drink; then go to sleep, if at night; if in the daytime, get up in an hour, dress, and be active for the remainder of the day. Suppose you sit still, in the damp clothing: in a few minutes chilliness is observed, the cold "strikes in," and next morning there is a violent cold, or an attack of pleurisy, or pneumonia, which if not fatal in a week, often requires weeks and months and weary years to get rid of. The short, sharp rule should be, if the clothing gets wet, change instantly, or work or walk actively, briskly, until perfectly dry.

